# A Step toward Tuberculosis Elimination in a Low-Incidence Country: Successful Diagnosis and Treatment of Latent Tuberculosis Infection in a Refugee Clinic

**DOI:** 10.1155/2016/7980869

**Published:** 2016-02-24

**Authors:** Elissa Rennert-May, Elisabeth Hansen, Toktam Zadeh, Valerie Krinke, Stan Houston, Ryan Cooper

**Affiliations:** ^1^Infectious Diseases, Faculty of Medicine and Dentistry, University of Alberta, Edmonton, AB, Canada T6G 2G3; ^2^Edmonton TB Clinic, Edmonton, AB, Canada T6G 2J3; ^3^New Canadians Clinic, Edmonton, AB, Canada T5M 3Z7; ^4^University of Alberta School of Public Health, Edmonton, AB, Canada T6G 1C9

## Abstract

*Objectives.* Approximately 65 percent of tuberculosis (TB) cases in Canada each year occur from reactivation in foreign-born individuals. Refugees are at high risk after immigration. Routine screening of this population for latent TB infection (LTBI) is generally considered infeasible. We evaluated the outcome of LTBI screening and treatment amongst refugees.* Methods.* Government-sponsored refugees in Edmonton are seen at the New Canadians' Clinic and screened for TB and LTBI. We reviewed records of patients between 2009 and 2011. Completeness of initial assessment, diagnosis of latent infection, and completion of LTBI treatment were evaluated. Treatment for LTBI was offered when patients had a positive Tuberculin Skin Test (TST) and risk factors for progression to TB. An Interferon-Gamma Release Assay (IGRA) was performed on all other TST positives; treatment is only offered if it was positive.* Results.* 949 refugees were evaluated. 746 TSTs were read, with 265 positive individuals. IGRA testing was performed in 203 TST positive individuals without other TB risk factors; 110 were positive. LTBI treatment was offered to 147 of 151 eligible patients, 141 accepted, and 103 completed a treatment course.* Conclusion.* We observed high proportions of patient retention, completion of investigations, and treatment. This care model promises to be a component of effective TB prevention in this high-risk population.

## 1. Introduction

Although tuberculosis (TB) remains an important cause of global mortality [1], most developed countries have seen significant declines in TB incidence over the past two decades [2]. In Canada, TB incidence had decreased to less than 5 cases per 100,000 persons by the 1990's, with the majority occurring in the foreign born [3]. Worldwide, the goal for TB rates in low-incidence countries such as Canada is less than 10 cases per million people per year by the year 2035 [4]. Unfortunately, the current annual reduction of TB in Canada is approximately half of the federal government's target, and Canada is unlikely to reach TB elimination targets on time. Thus, there is a need for more intensive and focused efforts in order to meet the goal of eventual TB elimination [5].

As is the case in the United States [6], the foreign-born individuals account for most new TB cases in Canada (64 percent of new TB cases in 2012) [3]. Most of these cases result from reactivation of latent TB infection (LTBI), as immigrants and refugees are screened for active TB disease prior to leaving their country of origin [7]. Effective strategies for the detection and treatment of LTBI amongst the foreign-born individuals would appear to be an essential component of any national effort toward TB elimination in low-incidence countries such as Canada [8]. However, this represents an enormous public health undertaking, which anticipated to require extensive resources [9]. In 2014, most immigrants to Canada originated from regions of the world highly endemic for TB, where infection prevalence exceeds one-third of the population [10, 11].

To improve efficiency, LTBI screening programs should be targeted toward groups with high prevalence of infection or risks for reactivation. Refugees are at particularly elevated risk, even compared to other classes of immigrants, both for LTBI and for reactivation to active disease once latently infected [12]. Proposed reasons for this include poorer overall health status, higher rates of HIV coinfection, and recent TB infection related to crowded living conditions in refugee camps [13]. Of all new residents in Canada in 2014, 8.9 percent were classified as refugees and 2.9 percent were government-sponsored refugees [10].

Postarrival screening programs for LTBI in immigrant and refugee populations are considered difficult to implement, compromised by practical difficulties in finding and engaging them after arrival and low reported treatment acceptance and completion [12, 14]. In Canada, there exists no formal, systematic, mandatory postarrival screening program for LTBI in immigrants [15], in spite of the recognition that, in order to eliminate TB from low-incidence countries, groups at high risk for reactivation will need to be successfully targeted for LTBI screening and treatment [8, 16, 17]. A recent Canadian publication highlighted this gap between a widely accepted goal and actual policy and practice and advocated for creative strategies to enable expanded implementation of LTBI testing and treatment in high-risk immigrants [12, 18].

In Edmonton, we recently developed an enhanced TB screening program for government-sponsored refugees after arrival. Prior to this, no systemic screening of refugees for LTBI was in place. The new program introduced a formal process for enhanced LTBI screening that included several interventions simultaneously. Specifically we introduced prompt evaluation within two weeks of arrival, integration of TB services with a general refugee health clinic, and placement of a Tuberculin Skin Test (TST) in most refugees, when indicated. We sought to evaluate this screening program and hypothesized that enhanced LTBI screening would lead to improved detection of LTBI and better uptake of LTBI treatment and ultimately decreased reactivation of TB disease.

## 2. Methods

In Edmonton, government-sponsored refugees are boarded in a reception house immediately upon arrival until a settlement worker locates more permanent housing, a process that takes approximately two weeks. Refugees receive prompt medical evaluation in the New Canadian's Clinic (NCC), located within the reception house while they are boarding there. A wide variety of medical services including assessment for LTBI, psychological support, screening for HIV, viral hepatitis, and parasitic infections are offered. Almost none of the refugees speak English as their first language but telephonic and in-person interpreters are available. All diagnostic tests and treatments for TB and LTBI were offered free of charge, and transportation subsidies such as taxi chits were offered. Prior to initiation of screening for LTBI, participants were informed that this was not a mandatory requirement and would have no impact on their immigration status.

Routine screening for active TB with symptom inquiry, chest radiograph (CXR), and sputum culture for those with an abnormal CXR occur at the first NCC visit and are integrated with the above medical care. Refugees with symptoms of active TB or a history of previous TB are referred expeditiously to a TB physician for further assessment of disease activity without waiting for further assessment of LTBI. Active TB is generally diagnosed within eight weeks of arrival to Canada.

All refugees aged six months to 50 years at the NCC without a history of treatment for active TB receive a TST, regardless of the presence of additional risk factors for progression from LTBI infection to active disease. If the TST is positive, a sputum sample (in those older than 6 years) is submitted for mycobacterial direct stain and liquid culture (BACTEC MGIT 960 System, Becton Dickinson 2006), and a CXR is performed. For those refugees older than 50 years, screening for LTBI with TST is only indicated if high-risk medical conditions including HIV, diabetes or chronic kidney disease are present (see Table 1 in [16]). A settlement counselor, fluent in the language of the refugee, helped guide clients to laboratory and medical appointments when necessary.

Patients aged five to 50 with a positive TST and no high-risk lung scarring on radiograph and no risk factors for progression to active TB were offered Interferon-Gamma Release Assay (IGRA) testing at no cost to the refugee. The use of sequential TST/IGRA testing in this group of refugees without additional risk factors for progression to active TB was an attempt to improve the cost-effectiveness of our approach in this population with high rates of previous BCG vaccination [19, 20]. LTBI treatment was then offered to IGRA-positive individuals. Patients with a positive TST and abnormalities on CXR or medical risk factors for reactivation were assessed by the TB physician and usually offered LTBI treatment, without further testing with an IGRA. At the NCC, the first-line treatment regime is nine months of daily isoniazid (INH) while four months of daily rifampin (RIF) was an alternative usually reserved for those demonstrating intolerance to INH. In summary, refugees who had a positive TST plus additional risk factors for progression to active TB or a positive IGRA were offered LTBI prophylaxis.

In the program, the tuberculin skin test is performed using the Mantoux method, utilizing 5 U of tuberculin (Pasteur institute). All TSTs are read by specially trained public health and/or TB nurses. We used the QuantiFERON Gold-in-Tube (QFT-GIT, Cellestis Ltd., Carnegie, Australia) which has a cutoff value for positivity of 0.35 IU/mL, as per the manufacturer's recommendation.

For this current evaluation, we reviewed patient charts on all patients seen at the NCC between January 1, 2009, and December 31, 2011. Demographic and clinical factors were extracted, as were proportions of patients who completed testing and treatment. We defined treatment acceptance as consent to take medication and starting one dose of medication. We defined LTBI treatment completion as having finished nine months of INH within 12 months of starting or completing four months of RIF within a six-month time period.

Ethics approval was granted from the University of Alberta Ethics Review Board.

Categorical variables were analyzed using Chi-square analyses and continuous quantitative variables were analyzed using student *t*-tests. All statistics were carried out using the program StatPlus (year 2009).

When comparing categorical variables, Sub-Saharan Africans were compared to all other regions combined together as they comprised more than half of the total participant group.

Vigorous efforts were made to retain patients receiving care for LTBI but, as is the case for all LTBI patients in Alberta, there was no active follow-up for the detection of TB. All refugee files were cross-referenced with the Alberta TB program to determine if any refugees were subsequently reported to provincial TB services with active disease.

## 3. Results

During the three-year interval between 2009 and 2011, 949 refugees were evaluated at the Edmonton NCC (Table 1). Adequate records were available for review in 948.

**Figure 1:**
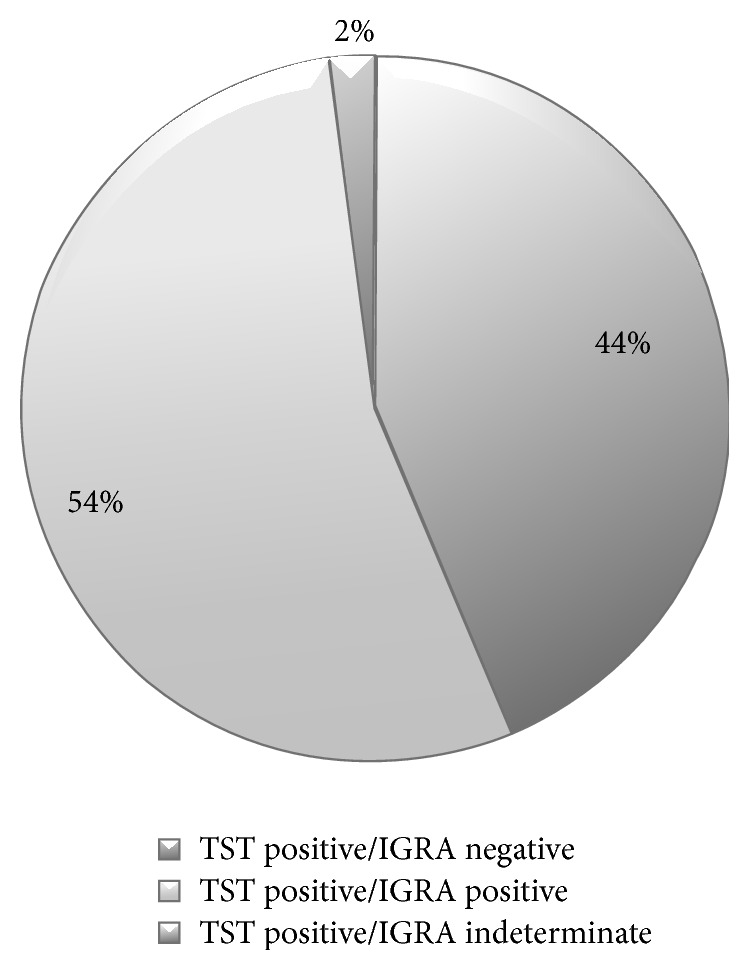
IGRA results following positive TSTs (*N* = 203).

Every patient seen at least once at the NCC received an initial assessment by a TB nurse (100%). Of these, 769 met criteria for TST (i.e., no active TB or a history of previous treatment, additional risk factors for progression to active TB, and six months to 50 years of age). In 746 patients (97% of those who met TST testing criteria) TSTs were successfully planted and read. Of those, 265 (36%) were positive. IGRA testing was performed in 203 TST positive individuals who did not exhibit other risk factors for progression to active disease. Of these, 110 (54%) were positive (Figure 1). Sub-Saharan Africans were more likely to have positive IGRA than refugees from other regions (75/105 (71%) versus 35/93 (38%) *p* < 0.005). BCG vaccination history was present in most refugees but individual patient level data was not available.

**Figure 2:**
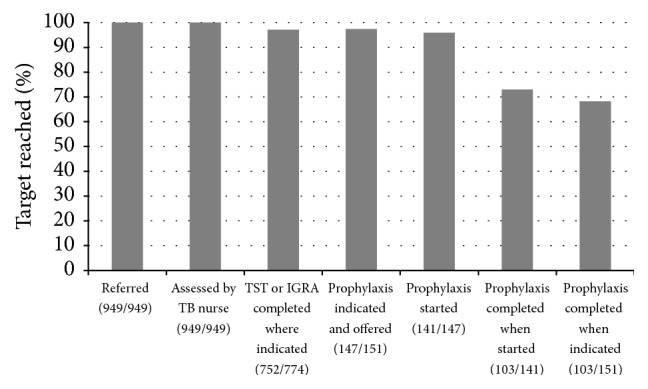
Cascade of care for refugees referred and followed at the New Canadians Clinic.

Of the 949 refugees, 151 were considered eligible for LTBI treatment. Of those 151 individuals, 110 were TST and IGRA positive and the other 41 were TST positive with a risk factor for TB reactivation. Of the 151 patients, two were lost to follow-up and two moved out of the region before treatment could be offered. Therefore, LTBI treatment was offered to 147 (Table 1). One hundred and forty one patients (96%) agreed to start treatment and 103 (73%) of those who started treatment completed an entire course of LTBI therapy. Therefore, amongst all participants for whom treatment of LTBI was considered indicated, 103 of 151 (68%) completed treatment (see Figure 2). Statistically significant correlates of nonadherence to therapy once started include young age (*p* = 0.039) and Sub-Saharan African origin (*p* < 0.005) (Table 2). Only 64% of Sub-Saharan African refugees starting preventative TB treatment completed it.

Treatment with INH was started in 122 patients: 13 were switched to RIF and INH was completed by 81 (74%). RIF started in 17 patients was completed by 12 (71%). Of the 13 patients started on INH who were subsequently switched to RIF due to adverse events or intolerances, 9 (69%) went on to complete the course. There were no significant differences in completion for those taking INH versus RIF (*p* > 0.1).

In the group of noncompleters, of the 32 patients started on INH, 21 (66%) completed less than three months and 14 (44%) completed less than one month. Of the 5 patients started on RIF, three (60%) completed less than 2 months.

Three of the screened refugees were found to be symptomatic during evaluation by the NCC nurse. They were all diagnosed with active TB (two with pulmonary TB and one with lymph node TB). A fourth patient, initially asymptomatic, was found to have a reactive TST (11 mm) on screening but a negative IGRA (0 IU/mL) and so was not offered preventative treatment; however, within a year of arrival he presented to a primary care provider with pulmonary TB.

## 4. Discussion

We found that a systematic program of screening and treatment of LTBI in refugees as part of a comprehensive refugee clinic was feasible and more effective than what has been previously reported in the literature. Our program resulted in high treatment completion (73%) and retention for each step in the screening process (>90%), as demonstrated in our “cascade of care” (Figure 2). Further, high (>90%) treatment acceptance in these refugees was observed. Prior to the introduction of this program, no systemic way to screen or identify LTBI amongst postarrival refugees existed. Though difficult to quantify how many refugees were offered LTBI treatment as a result of passive, nonprogrammatic detection efforts prior to the implementation of our program, we estimate this number to have been very low with treatment completion being even lower. Enhanced screening and treatment for LTBI amongst refugees could be expanded as an important tool for reducing reactivation TB in low-incidence countries.

Detecting and treating LTBI amongst refugee populations is a multistep process, with chance for attrition at every step in the “cascade of care” (Figure 2). How many immigrants potentially eligible for LTBI screening are actually identified and how many subsequently undergo TST or IGRA re often not clear in other published reports of postarrival immigrant screening for LTBI. Most previous studies report on acceptance and completion in individuals after they have already been successfully engaged and screened. Our population included 100 percent of government-sponsored refugees arriving in Edmonton.

Once screened and deemed eligible for LTBI treatment, acceptance (i.e., agreeing to take the treatment) and completion proportions observed in our study compare favorably to those reported in the literature. A review of LTBI detection and treatment in foreign born and low socioeconomic populations showed average initial acceptance rates ranging from 26 to 91 percent [21]. Reported rates of treatment acceptance in difficult to engage populations such as injection drug users, incarcerated patients, and refugees are generally low, between 50 and 80 percent [22].

A previous Canadian study examining treatment acceptance and completion in refugee claimant populations in Montreal, Quebec, is one of very few studies completed exclusively with a refugee population. They demonstrated an acceptance rate of 77 percent [23].

A systematic review and meta-analysis of LTBI screening in immigrants arriving into countries of low TB incidence found substantial loss at every step of the cascade of care and ultimately only 32 percent of those found to have LTBI actually completed treatment with prophylaxis [24]. The low rate of completion was felt to be in part due to a lack of linguistic and cultural support for the immigrants. In the above-mentioned study examining a specifically refugee population in Canada, only 49 percent of participants completed at least six months of treatment with INH [23].

The refugee population in our study may have been more likely to accept and begin treatment for LTBI as medical assessment was an integrated component of a comprehensive new arrivals program. Previous studies have demonstrated that a convenient clinic schedule is associated with higher rates of acceptance for treatment of LTBI [22].

We found that almost half of those with reactive TSTs were subsequently negative on further testing with IGRA. A relatively high rate of discordance between TST and IGRA has been well documented in populations with previous BCG vaccination [25]. The Centers for Disease Control (CDC) in the United States recommends the use of either the IGRA or TST as a screening tool for LTBI [26]. However, there is some evidence that the IGRA has better specificity than the TST in predicting future reactivation TB [27]. To the degree that this is the case, the IGRA in any diagnostic algorithm is likely to reduce the number of patients requiring LTBI treatment and follow-up and may improve cost-effectiveness if it allows programmatic efforts to be targeted to those most likely to progress to active TB [28]. Using the IGRA instead of a TST would likely further streamline the assessment process and reduce the number of required clinic visits resulting in a more patient centered approach. On the other hand, we acknowledge that an unknown number of individuals with latent infection may have been misclassified on the basis of false-negative IGRA results [29]. This was exemplified in our cohort by a TST positive but IGRA negative case that subsequently developed active disease after arrival to Canada. Certainly, the use of an IGRA following a TST remains controversial and is a practice that, while regularly used in Alberta, is not the standard of care across Canada. Our policy of not routinely offering LTBI treatment to patients without additional risk factors for progression to active TB and who had a negative IGRA following a positive TST had been previously recommended by contemporary expert opinion [30]. Our results did indicate that patients who originated from countries with the highest rates of TB were more likely to have a positive IGRA after a positive TST. This implies that there may be little utility to doing both the TST and an IGRA in this subset of patients, and clinic policy on this issue is being reviewed.

In contrast to previous studies, we found no difference in completion between nine months of INH and four months of RIF in our study population although the number started on RIF was low [14, 16, 31]. It is possible that, with close follow-up provided in our program via settlement counselors and nurse visits with regular refills, attrition in both arms was limited, making any potential difference in completion difficult to detect. Though we found no difference in completion between INH and RIF, it should be noted that while 13 people switched from INH to RIF because of adverse events (such as hepatotoxicity and rash), only one person switched from RIF to INH. This is consistent with previous literature demonstrating better tolerability and lower cost of RIF treatment over INH [32].

In conclusion, our study has demonstrated that early integration of LTBI screening into refugees' medical care can result in very high proportions of patient retention as well as excellent proportions of acceptance and completion of LTBI treatment. Moreover, three cases of active TB were identified during this screening process. These results are the benefit of a well-structured and evidence-based health service program for refugees. The cost-effectiveness of this program should be evaluated in the future. Additionally, while the program did attempt to be person centered by offering all services free of charge, convenient appointment times, translators, and funded transportation, future studies could consider the use of focus groups to look at the impact of this program on an individual patient level. The NCC program may provide a useful model for future programs targeting high-risk LTBI populations with the potential to further reduce the pool of individuals at risk of TB reactivation and advance progress toward TB elimination.

## Figures and Tables

**Table 1:** Demographic information.

Demographics	*N* (%) or mean (StDev) total *N* = 949	Offered prophylaxis *N* (%) or mean (StDev) total *N* = 147
Age	23.7 (16.7)	29 (15.1)
Gender		
Male	474 (49.9)	75 (51)
Region		
Sub-Saharan Africa	433 (45.6)	88 (59.9)
Middle East	251 (26.4)	20 (13.6)
South Asia	118 (12.4)	25 (17)
East and Southeast Asia	88 (9.3)	11 (7.5)
Others	59 (6.2)	3 (2)

**Table 2:** Of those patients accepting treatment for latent tuberculosis infection, this is a comparison of completers versus noncompleters of tuberculosis prophylaxis by age, gender, and country of origin.

Demographics	Completed prophylaxis *N* (% of total row value) or mean (StDev) *N* = 103	Did not complete prophylaxis *N* (% of total row value) or mean (StDev) *N* = 38	*p* values
Age	29.9 (15.6)	23.6 (16.7)	<0.05
Gender			
Male	54 (73)	20 (27)	>0.05
Female	49 (73)	18 (27)	
Region			
Sub-Saharan Africa	54 (64)	30 (36)	<0.05
Middle East	12 (67)	6 (33)	
South Asia	25 (100)	0 (0)	
East and Southeast Asia	11 (100)	0 (0)	
Others	1 (33)	2 (67)	
